# The complementarity-determining region sequences in IgY antivenom hypervariable regions

**DOI:** 10.1016/j.dib.2017.07.005

**Published:** 2017-07-08

**Authors:** David Gitirana da Rocha, Jorge Hernandez Fernandez, Claudia Maria Costa de Almeida, Claudia Letícia da Silva, Fabio Carlos Magnoli, Osmair Élder da Silva, Wilmar Dias da Silva

**Affiliations:** a Laboratório de Biologia do Reconhecer (LBR), Centro de Biociências e Biotecnologia (CBB), Universidade Estadual do Norte Fluminense Darcy Ribeiro (UENF), Alberto Lamego Avenue, 2000 – Campos dos Goytacazes, Postal Code 28013-602 Rio de Janeiro, Brazil; b Laboratório de Química e Função de Proteínas e Peptídeos (LQFPP), Centro de Biociências e Biotecnologia (CBB), Universidade Estadual do Norte Fluminense Darcy Ribeiro (UENF), Alberto Lamego Avenue, 2000 – Campos dos Goytacazes, Postal Code 28013-602 Rio de Janeiro, Brazil; c Laboratório de Sanidade Animal (LSA), Centro de Ciências e Tecnologias Agropecuárias (CCTA), Universidade Estadual do Norte Fluminense Darcy Ribeiro (UENF), Alberto Lamego Avenue, 2000 – Campos dos Goytacazes, Postal Code 28013-602 Rio de Janeiro, Brazil; d Laboratório de Imunoquímica, Instituto Butantan, Vital Brazil Avenue, 1500 – City of São Paulo, Postal Code 05503-900 São Paulo, Brazil

**Keywords:** PCR, Sequencing, Modeling of biomolecules

## Abstract

The data presented in this article are related to the research article entitled "Development of IgY antibodies against anti-snake toxins endowed with highly lethal neutralizing activity" (da Rocha et al., 2017) [1]. Complementarity-determining region (CDR) sequences are variable antibody (Ab) sequences that respond with specificity, duration and strength to identify and bind to antigen (Ag) epitopes. B lymphocytes isolated from hens immunized with *Bitis arietans* (Ba) and anti-*Crotalus durissus terrificus* (Cdt) venoms and expressing high specificity, affinity and toxicity neutralizing antibody titers were used as DNA sources. The VLF1, CDR1, CDR2, VLR1 and CDR3 sequences were validated by BLASTp, and values corresponding to IgY V_L_ and V_H_ anti-Ba or anti-Cdt venoms were identified, registered [*Gallus gallus* IgY Fv Light chain (GU815099)/*Gallus gallus* IgY Fv Heavy chain (GU815098)] and used for molecular modeling of IgY scFv anti-Ba. The resulting CDR1, CDR2 and CDR3 sequences were combined to construct the three - dimensional structure of the Ab paratope.

## **Specification Table**

**Table:** 

Subject area	*Biology*
More specific subject area	*Immunology (antibodies biotechnology)*
Type of data	*Table, images and figures*
How data was acquired	*Conducting the immunization of hens with specific venoms, to produce antivenom sera.* *B lymphocytes were separated from serum samples, and specific mRNAs were transformed into cDNAs and amplified.* *The cDNAs were analyzed in sequencing procedures and BLASTp.* *The modeling analysis was made.*
Data format	*Raw and analyzed data*
Experimental factors	*The specific cDNA collection, related to late antibody production, sequencing and analyzing the protein product of the correct reading frame, in order to produce viable models of minibodies.*
Experimental features	*The beginning process to analysis and produce antivenom scFv for therapeutic purposes.*
Data source location	*Campos dos Goytacazes, Rio de Janeiro – Brazil (-21.7605295,-41.2948636)*
Data accessibility	*The main data is available in this article.* *The sequences used to perform the presented model are available in:* *GU815098 –*https://www.ncbi.nlm.nih.gov/nuccore/gu815098 *GU815099 –*https://www.ncbi.nlm.nih.gov/nuccore/gu815099

## Value of the data

• Data on hypervariable regions of IgY antivenom expressing high specificity-avidity-affinity-toxins neutralizing allow isolation avian CDRs mammals counter party.
• The IgY CDRs DNA basing pares and amino acid sequences formed linear and three-dimensional molecular structures similar to IgG.
• An entire immunoglobulin molecular model was constructed by substituting the CDR-1, CDR-2 and CDR-3 in human IgG by the chicken corresponding CDRs.
• This feasibility open up an alternative platform of antitoxins Abs modeling fused fragments variable Fv.

## 1 Data

Hens were immunized with Cdt or Ba venoms. Punctual interferences of horse immunization schedules with lower immunizing venom doses and an increased number of immunizations interspaced with longer periods and meticulous antibody quality evaluations [1], [2], [3] were transferred to immunize hens [1]. More specific antibodies endowed with higher affinity and toxin neutralizing potency were developed. B lymphocytes recovered after the 7th immunization served as the sources of DNA. mRNA was isolated and sequenced using chicken nucleotide sequence (5′-3′) primers [4] (Table 1).

**Table 1:** IgY scV_H_ and scV_L_ primers sequences [4].

**Primers**	**Sequences**
**VLF1**	GACTCAGCCGTCCTCGGTGTCAG
**VLR1**	TGATGGTGGCGGCCGCATTGGGCTG
**VHF1**	CTGATGGCGGCCGTGACGTTGGAC
**VHR1**	CCGCCTCCGGAGGAGACGATGACTTCG

The IgY antivenom CDR sequences were determined from immunized B cells by amplification of VL and VH chain genes, preparation of IgY chain double-strand cDNAs, and clonal selection and sequencing of hyper-variable regions of IgY genes. The obtained sequences were submitted to Blast analysis, and the gene sequences corresponding to CDR1, CDR2 and CDR3 in IgY V_L_ and V_H_ anti-*Bitis arietans* (Ba) or *C. d. terrific* sequences were amplified. The encoding gene sequences and the translated amino acid sequences served to construct an IgY 3D molecule model.

## 2 Experimental design, materials and methods

### 2.1 Amplification of IgY H and L domains

**Fig. 1:**
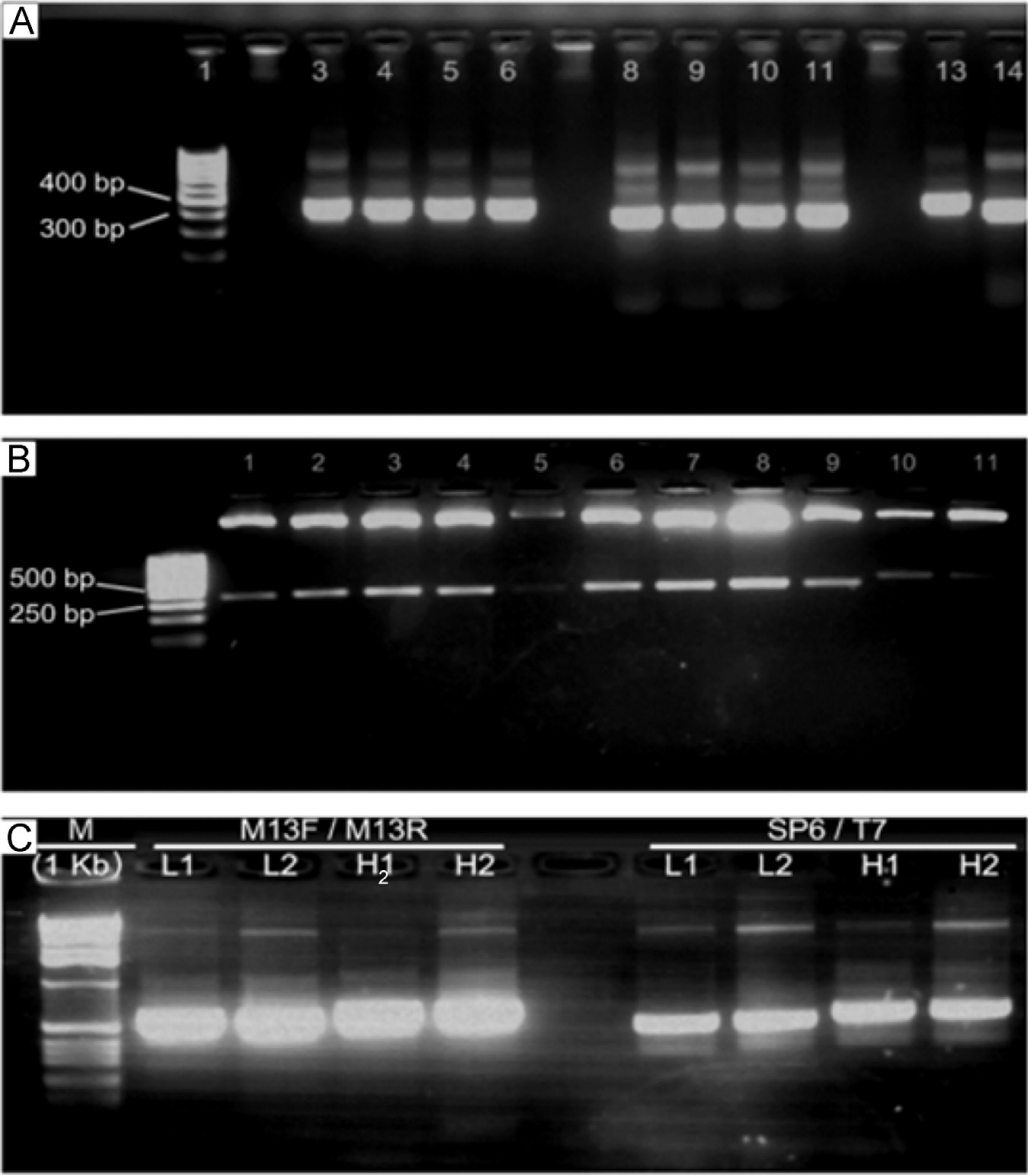
Amplification of IgY H and L domains. (**A**) Domains IgY H (**lanes 3–6**) and L (**lanes 8 to 11**) from hens cDNA; (**B**) digestion assay using EcoRI enzyme to verify the positivity of amplified plasmids (pGEM T easy). L domain (**lane 1–9**) and H domain (**lanes 10 and 11**); (**C**) amplification test before the sequencing procedures, with M13F/M13/R and SP6/T7 sequencing primers. L – Light IgY chain, and H – Heavy IgY chain.

mRNA was isolated from B cells after the 7th immunization → Insertion into the pGEM T easy cloning vector → Transformed into DH5α *Escherichia coli →* FvL and FvH insert positive clones were purified with M13F/M13R primers → The nucleotide sequences were analyzed by comparing with BLASTp for homology comparisons → The products were amplified by RT-PCR using the M13F primer for direct reading and the M13R primer for inverse reading → The products were cloned twice with “*Big Dye Terminator Cycle Sequencing* → The selected frames shared homologies with mammalian IgG (50%) and IgE (60%) as determined by an analysis using the NCBI GenBank database. The IgY H domains (lanes 3–6) and L domains (lanes 8–11) from the hen cDNA were re-amplified and cloned into the pGEM T Easy vector (lanes 1–9 and lanes 10 and 11, respectively) → Amplifications of the L, V_L_, H, and V_H_ variable IgY regions were then performed with the M13F/M13R and SP6/T7 sequencing primers (Fig. 1).

**Fig. 2:**
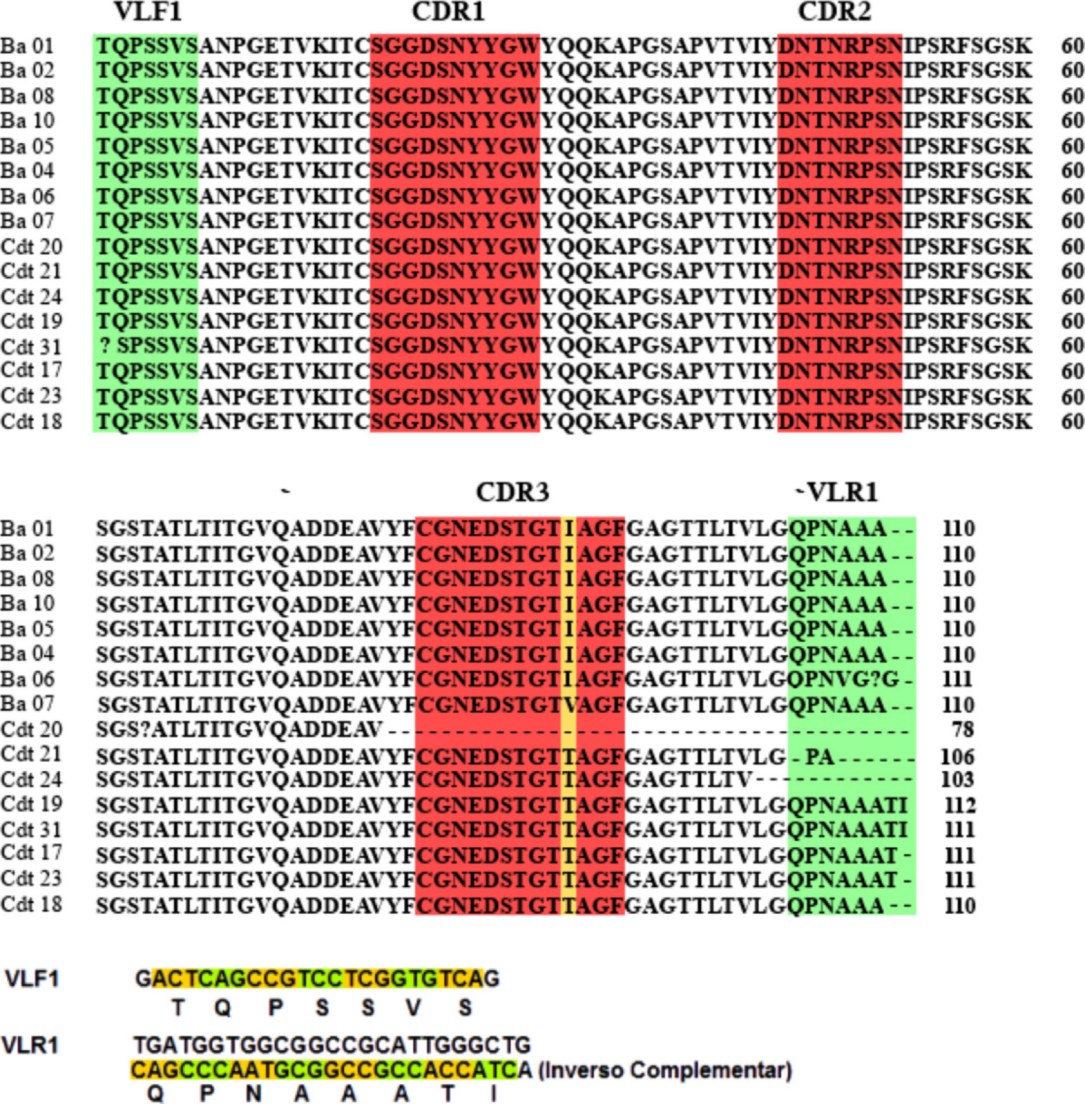
IgY anti-*Bitis arietans* (Ba) and *Crotalus durissus terrificus* (Cdt) venoms light chain variable sequences validated by BLASTp.

The light chain variable sequences of the IgY anti-Ba and anti-Cdt venoms were validated by BLASTp. The VLF1 primer (*in green color*), CDR1, CDR2, CDR3 (*in red color*), and VLFR1 primer (*in green color*) sequences are indicated (Fig. 2).

**Fig. 3:**
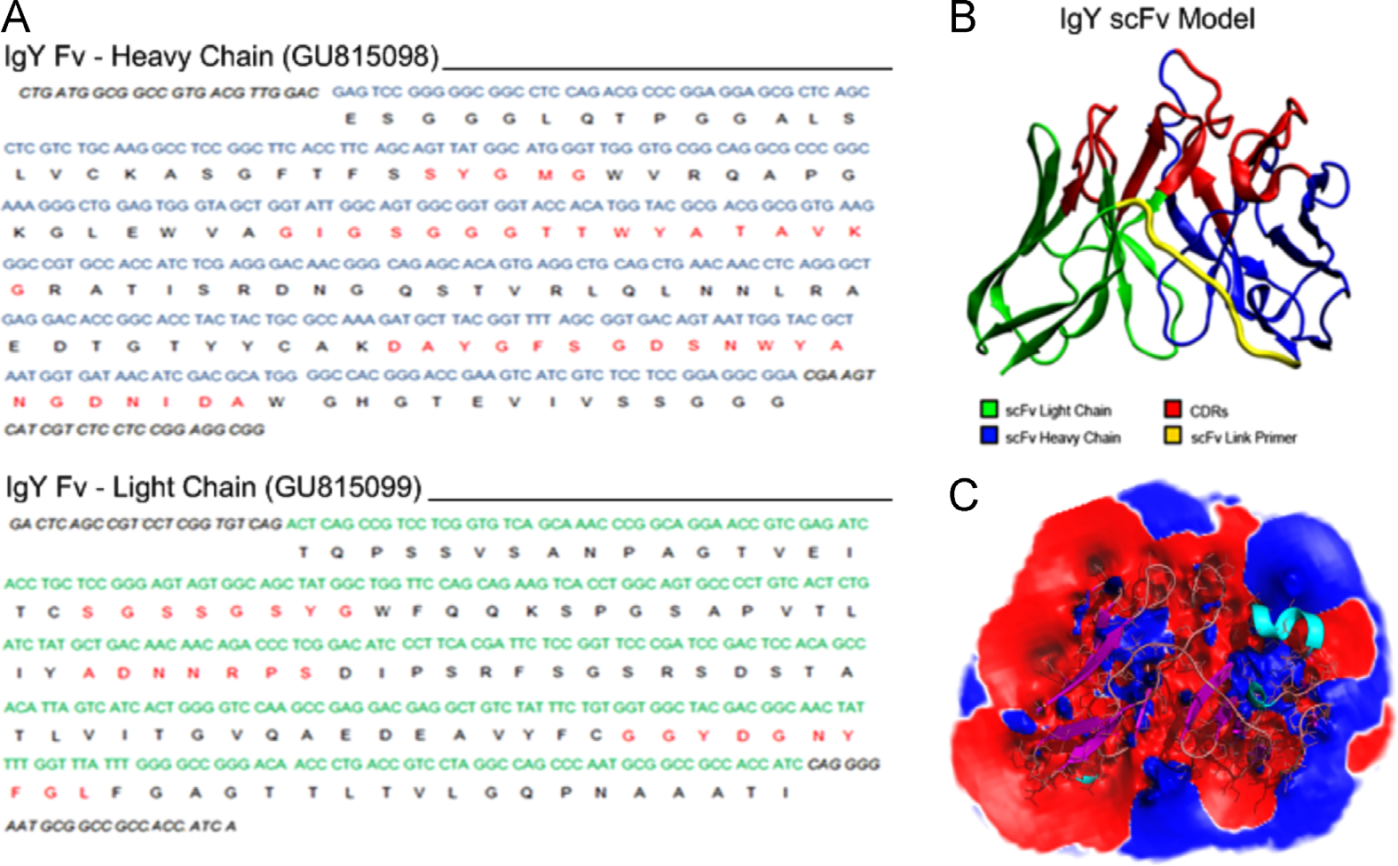
(A) Sequences H and L of a scFv against a protein of *Bitis arietans* venom. Red sequences indicate the CDRs; (B) modeling of scFv. Red sequences indicate CDRs. Yellow color sequences comprise ligation segments though introduced six amino acid residues aiming adequate rearrangement of 3D molecule; (C) Hydrophobic (blue color) and hydrophilic (red color) domains of scFv model. The paratope region (comprising CDRs) is allocated on hydrophilic side.

The resulting amino acid sequences were used for modeling IgY scFv are in Fig. 3.

**Fig. 4:**
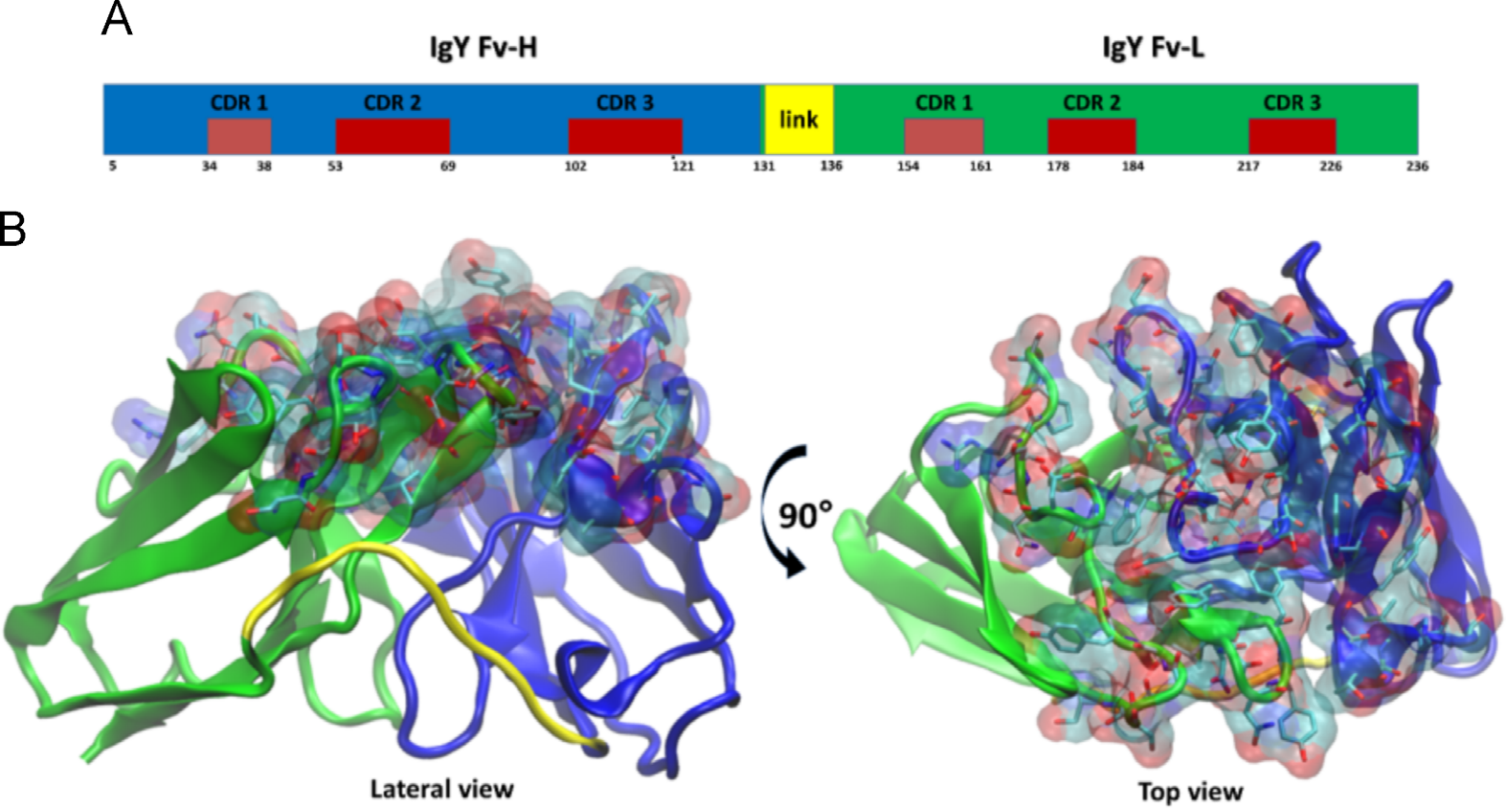
Antivenom IgY scFv map indicating the position of each CDR (red) and the link primer (yellow); (B) simulation of contact with antigen. The base of Light (green) and Heavy (blue) chains regions are rich on β-strands, but not the top (paratope). The yellow sequence is the link primer. Lateral and top views.

The human IgG 3D molecular model combined with the IgY-CDRs ribbon diagram was further engineered to show putative molecular distortions upon binding to the specific antigen (Fig. 4).
