# Supplementary material for: The complementarity-determining region sequences in IgY antivenom hypervariable regions
Source: Data Brief. 2017 Jul 8;13:717–22. doi: 10.1016/j.dib.2017.07.005 (PMC5512210; doi:10.1016/j.dib.2017.07.005)
Supplement: Supplementary file 1 — Supplementary material [file mmc1.docx]

**CONFLICT OF INTEREST**

The authors have no finantial concflicts of interest.


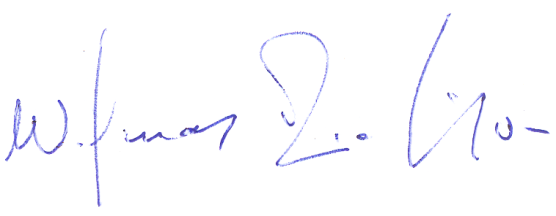


Wilmar Dias da Silva – Correspondent Author.
